# Influence of personality traits and clinical role models on changes in medical students’ attitudes toward psychiatry: a prospective moderation analysis

**DOI:** 10.1038/s41598-026-62087-0

**Published:** 2026-07-16

**Authors:** Hajir Fajwani, Tamadhir Al-Mahrouqi, Ali Al-Harthi, Mohammed Al-Alawi

**Affiliations:** 1 Psychiatry Residency Training Program, Medical Specialty Board, Muscat, Oman; 2 Department of Behavioral Medicine, College of Medicine and Health Sciences, Sultan Qaboos University, Muscat, Oman

**Keywords:** Attitudes towards psychiatry, Personality, Role models, Medical students, Social learning theory, Health care, Psychology, Psychology

## Abstract

Oman faces a critical shortage of psychiatrists, highlighting the need to better understand determinants of medical students’ career attitudes toward psychiatry. Utilizing Social Learning Theory and Situational Strength Theory, this study examined how attitudes toward psychiatry evolve during clinical training through the interaction between individual characteristics (personality traits) and contextual influences (clinical learning environment and role models). The study aimed to determine whether personality traits and exposure to clinical role models moderate changes in students’ attitudes during the psychiatry clerkship. A prospective single-group quasi-experimental pre-post study was conducted among 180 undergraduate medical students at Sultan Qaboos University during an eight-week psychiatry clerkship. Attitudes toward psychiatry and personality traits were assessed using the Attitudes Toward Psychiatry-30 (ATP-30) and Big Five Inventory-44 (BFI-44) scales. Moderation analyses were performed using Hayes’ PROCESS macro to evaluate interaction effects between baseline attitudes, personality traits, and perceived role-model influence. Attitudes toward psychiatry improved significantly following clerkship exposure (mean ATP-30 score increased from 105.66 to 110.38; *p* < .001). Personality traits did not significantly moderate attitude change. However, perceived resident role-model quality demonstrated a significant interaction with baseline attitudes (b = − 0.953, *p* = .004), with the greatest improvement observed among students with initially less favorable attitudes toward psychiatry. Prior personal contact with mental health services was independently associated with lower post-clerkship attitude scores (b = − 4.553, *p* = .047). Psychiatry clerkships were associated with improved student attitudes, with role-model mentorship appearing more consequential than stable personality characteristics. As improved attitudes do not consistently translate into specialty recruitment, workforce strategies should move beyond passive clinical exposure toward structured mentorship, targeted career engagement, and culturally responsive psychiatric training models.

**Supplementary Information:**

The online version contains supplementary material available at https://doi.org/10.1038/s41598-026-62087-0.

## Introduction

Oman, like many nations globally, faces a critical shortage of psychiatrists within its healthcare system. The World Health organization (WHO) generally recommends a benchmark of 1 psychiatrist per 10,000 people. Oman as of 2020 has a density of 1.54 psychiatrists per 100, 000 people^1^, just 15% of the global target. Such a severe workforce deficit places immense strain on mental health services and limits the population’s access to timely, evidence-based psychiatric care. These deficits, if maintained, will inevitably stretch resources thin, leading to increased rates of long-term institutionalization and a rise in psychiatric morbidity and mortality^1^ t. In this context, many Omanis suffering from mental disorders often first visit a spiritual/traditional healer rather than a psychiatrist. This is due to several reasons, including limited accessibility for psychiatric services due to geographical and logistic barriers, societal stigma, and the predominating cultural beliefs^2^. Stigma surrounding mental illness acts as an additional burden; it affects not only those suffering from mental disorders but also their families and caregivers, while also straining the entire mental health care infrastructure, including psychiatrists, psychologists, and healthcare institutions^3^.

Furthermore, systemic inefficiencies and the perceived lack of clinical resolution in psychiatry contribute to a crisis of confidence in mental health services. Patients may disengage from psychiatric treatment, reinforcing reliance on traditional healing pathways and perpetuating societal stigma. For medical students who often cite the ability to effect tangible clinical improvement as a core motivator in specialty selection psychiatry’s association with chronicity and limited prognosis can be dissuasive. Prior research indicates that a perceived treatment inefficacy and poor patient outcomes are among the key deterrents for students considering psychiatry as a career^4^. When students observe a system defined by chronic management rather than resolution, the anticipated emotional burden discourages them from pursuing the field. This creates a self-perpetuating cycle where a lack of specialty appeal further depletes the workforce, ultimately compromising the future of psychiatric care in the Sultanate.

Within this strained system, both doctors and medical students agree that dealing with psychiatric patients is challenging. The stigma towards mental illness, both in society and within the medical field itself, negatively affects the judgment of medical students regarding psychiatry as their future career path. As fewer graduates choose the specialty, the workload on psychiatrists increases. To reduce this burden, we must motivate and encourage more medical students to specialize in psychiatry^5,6^.

This concern is echoed across the Eastern Mediterranean Region (EMR). A 2023 systematic review found that although medical students generally report positive attitudes toward psychiatry, this rarely translates into specialty choice^7^. The average psychiatrist density in EMR countries remains at just 1 per 100,000, in stark contrast to 8 or more per 100,000 in high-income countries. Studies from Sultan Qaboos University^8^ and the UAE^9^, have replicated these findings, revealing especially low interest among male students, despite positive exposure. A 2013 global review similarly highlighted this disconnect between attitude and career selection^10^.

Beyond the EMR, research around the world showed that students often reject psychiatry due to medical unrewardability, citing concerns that the discipline lacks a physiological focus and fails to fully utilize their medical training. Additional discouraging factors included a perceived low efficacy of treatments, poor patient prognosis, and low job satisfaction^11^.

Compounding these perceptions are concerns specific to psychiatric pharmacotherapy: students frequently report unease about the chronic nature of psychopharmacological management, in which treatment controls rather than resolves symptoms, and about prescribing medications with dependence potential^12^. Another increasingly relevant deterrent is the perceived psychiatrization of emotional distress; where the psychiatric field risks pathologizing ordinary human adversity and erodes individual’s capacity for autonomous coping^13^.

Furthermore, social pressures also play a decisive role; the low prestige of the specialty and negative pressure from peers and family undermine a student’s willingness to pursue the field^11^. These findings suggest that the effectiveness of a psychiatric rotation is determined by the quality of the clerkship, particularly in its ability in linking evidence-based medicine (EBM) to practice and applying preclinical medical knowledge. Moreover, the perceived attractiveness of the psychiatrist’s lifestyle, the positive impact psychiatric treatment has on patients’ lives, and the influence from role models play a vital part in combating this stigma and raising specialty appeal^7^.

This present study attempts to explore the role of personality traits and role models within psychiatry on the change in the attitude toward the specialty pre- and post-psychiatry rotation. This study is primarily based on social learning theory, which states that our attitude, in general, is shaped by the environment and personal qualities^14^.

Personality represents the internal arm of this interaction, defined as the differences in characteristic patterns of thinking, feeling, and behaving in individuals^15^. The Big Five Inventory operationalizes this construct through five traits: openness to experience, conscientiousness, extraversion, agreeableness, and neuroticism. Openness to experience is when an individual has imagination and active life, is curious, and is open to a variety of experiences. Conscientiousness is when the person is sensible, reliable, well organized, and goal oriented. Extraversion is an individual who is social, forceful, dominant, and has positive emotions. In agreeableness, they are cooperative and sympathetic toward others, moreover, they exhibit trust and altruism. In neuroticism they experience emotional instability and find it difficult to cope with their stress^16^. While global studies using the Big Five Inventory show conflicting results regarding which traits predict psychiatric interest, such as high openness in Sweden^17^, high extraversion and low conscientiousness in Finland^18^, or high agreeableness and openness but low extraversion in Croatia^19^– these inconsistencies point to the importance of individual differences and contextual determinants. Beneath these trait dimensions lies a deeper biological layer of affective temperament, which shapes emotional reactivity and has been linked to psychiatric outcomes across clinical populations^20^; however, exploring this level of analysis remains beyond the scope of this present study.

Beyond individual traits, the environment represents the second arm of this interaction, explained through Situational Strength theory, which proposes that the strength of a given situation can constrain the variance of an outcome, stifling the predictive effect of internal factors such as personality. A highly structured clinical rotation acts like a strong situation, where the impact of personality is moderated by external factors, most notably, exposure to role models. A role model is defined as a person who is admired and emulated for their behavior, attitudes, or career success^21^. A 2015 study from the University of Western Australia demonstrated that students who perceived their psychiatry supervisors as inspirational were more likely to pursue psychiatry^6^.

### Aim and objectives

Aim: Given the regional psychiatrist shortage, entrenched stigma, and the observed disconnect between attitude and specialty choice, this study aims to assess the moderating roles of personality traits and exposure to role models in shaping medical students’ changing attitudes toward psychiatry. By using a pre- and post-psychiatry rotation design among students at Sultan Qaboos University, this study seeks to identify which personality traits and environmental influences most significantly impact attitude change, thereby informing targeted interventions to enhance recruitment into psychiatry.

Objectives:

1. To describe the change in attitudes toward psychiatry.
2. To assess the moderating role of personality traits (Big Five Inventory) on attitude change.
3. To assess the moderating role of role models (Residents and Attendings) on attitude change.
4. To evaluate the strength of these moderations by adjusting for key covariates, including sociodemographic factors, academic performance, personal or family history of mental illness, and prior contact with mental health services.

Hypotheses:

1. The attitude of medical students will improve significantly following their psychiatric rotation.
2. Personality traits, namely openness and agreeableness, will significantly moderate positive attitude shifts.
3. Positive exposure to role models will significantly moderate positive attitude shifts.
4. The moderating effect of personality and role models will remain robust after adjustment for covariates.

## Methods

### Study design

This is a prospective single-group quasi-experimental pre-post study. conducted among medical students at the College of Medicine and Health Sciences at Sultan Qaboos University (SQU). The study population consisted of students completing their psychiatry rotation during the 2024–2025 academic year. Clinical training is centered at University Medical City (UMC) at Sultan Qaboos University Hospital (SQUH). SQUH is a tertiary referral center that manages approximately 10,000 outpatient visits per month. It serves a diverse patient demographic from both urban Muscat and rural governorates, providing a range of services from specialized tertiary care to primary care.

During the psychiatry rotation, students train across two sites:

1. Department of Behavioral Medicine at SQUH: The only department providing specialist tertiary psychiatric referrals in a general hospital. It offers services for general adults, geriatrics and child and adolescents, alongside liaison psychiatry and psychosocial intervention.

2. Al-Massarah Hospital: A specialized national psychiatric referral center providing General Adult Psychiatry, Geriatric Psychiatry, Child and Adolescent Psychiatry, and Forensic Psychiatry, as well as psychology, social work, rehabilitation services, somnology, physiotherapy, occupational therapy, and speech therapy.

### Study sample and sampling method

All medical students enrolled in the psychiatry clerkship were invited to participate via institutional email. Participation was voluntary.

### Consent procedure

Before starting the survey, the students received an online consent form via email inviting them to participate in the study. The online consent form explained the aims of the study, that their contribution to the study would be confidential, that their participation is based on autonomy, and that they could withdraw from the study at any time. Later, consenting students were invited to an online web questionnaire.

### Inclusion criteria

All medical students, regardless of their gender, doing their psychiatry clerkship in the academic year 2024–2025 and who can give their consent were eligible to participate in the study process of data collection.

### Exclusion criteria

Students who did not provide informed consent were excluded from participation. In addition, students who failed to complete both the pre-clerkship and post-clerkship questionnaires were excluded from the final analysis to ensure the validity of within-subject comparisons.

### Sample size

The G^*^Power 3.1i^®^ program was used to calculate the sample size. To achieve medium effect size of 0.15 based on F tests and multiple linear regression analysis considering the total of 5 predictors and three tested predictors, the required sample was 77. The following parameters were computed as well as the power level of 80.0%, a type-1 error of 5.0% (alpha = 0.05) and a 95% level of significance^22^.

Process of data collection.

### Pre-rotation survey

Consenting medical students before starting their psychiatry clerkship were invited to fill in a pre-psychiatry clerkship online questionnaire which will include:

1. Socio-demographic section.

2. Big Five Inventory Questionnaire.

3. Attitudes Towards Psychiatry Questionnaire (ATP-30).

### Post-rotation survey

After 8 weeks, on completion of their rotation in psychiatry, students were given a post-psychiatry clerkship online questionnaire which will include:

1. Attitudes Towards Psychiatry Questionnaire (ATP-30) once again to assess their attitude toward psychiatry post-clerkship.

2. Role model questionnaire post-psychiatry clerkship to assess the impact they have on attitude toward psychiatry.

### Outcome measure

#### Sociodemographic survey

This survey documented factors that could influence attitude toward psychiatry either positively or negatively, including participant’s age, gender, geographical stay, GPA (self-reported), whether they experienced any mental illness or have/had a relative with mental illness and whether they had personal experience or encounter with governmental or private mental health services. It will also contain a question that will be administered both pre- and post-psychiatry clerkship to explore whether psychiatry has been their first, second or third career choice.

#### Attitude toward psychiatry

The ATP-30 is a widely used 30-item questionnaire introduced by Burra et al.,^23^, with adequate validity and good internal consistency (Cronbach’s alpha = 0.874). It consists of 15 positively formulated and 15 negatively formulated items to minimize response bias. Respondents specify their agreement/disagreement with each item in terms of a five-point Likert scale (1 = agree strongly, 2 = agree, 3 = neutral/no opinion,4 = disagree, 5 = disagree strongly). The total score ranges from 30 to 150. To calculate the total score, positive items are reverse-scored so that an answer of 1 (agree strongly) equals 5 and an answer of 5 (disagree strongly) equals 1. Meanwhile, the score for negative items is taken as is. A cut-off score of 90 was used, where a global score of > 90 indicates an overall positive attitude, and a score of < 90 indicates a negative attitude, while a global score of 90 suggests a neutral attitude^24^. In the present sample, internal reliability for the ATP-30 was (Cronbach’s alpha = 0.86) for the pre-clerkship phase and (Cronbach’s alpha = 0.88) for the post-clerkship phase.

#### Big five inventory

It is a 44-item inventory that assesses a person on the Big Five Factors of personality. Every factor is then additionally divided into personality facets. The students are then supposed to write a number next to each item whether they agree/disagree in terms of a five-point scale ranging from 1 to 5, 1 being disagreed strongly to 5 being agreed strongly^25^. The instrument has demonstrated high internal consistency; reported Cronbach’s alpha coefficients in literature range from 0.73 for agreeableness to 0.80 for conscientiousness, extraversion, and neuroticism, with openness at 0.77^26^.

### Role model questionnaire

The influence of role models was examined by an agree or disagree question asking about whether the students encountered a role model that affected their attitudes toward psychiatry either positively or negatively. Those role models could either be residents, specialists, or consultants.

The questions included the following items, adapted from Balon et al.,^27^:

1. During my psychiatry rotation, psychiatry residents were good role models.

2. Attending psychiatrists during my psychiatry rotation were good role models.

### Statistical analysis

Descriptive analysis was conducted for socio-demographics and other variables. Mean and standard deviation (SD) was calculated for continuous variables; while for categorical variables, their frequency and percentage was presented.

The Hayes regression-based PROCESS macro was used to test for the moderation relationships. The PROCESS macro was selected because it offers the opportunity to determine the interaction effect by generating a series of plots that were later put together into a diagram or graph. The diagram further illustrated the conditional effect of X (pre psychiatry rotation ATP score) on Y (post psychiatry rotation ATP score), as a function of M (moderator variables including personality traits and role models represented as dummy variables). The moderating effects were thereafter examined using the regions of significance in accordance with the Johnson-Neyman technique. PROCESS is a better choice for research where the variables are all directly measured (e.g., in psychological settings that use hard data). All analyses were conducted using the Statistical Package for Social Sciences (SPSS) version 22.

Before analysis, the pre- and post-clerkship survey data were matched to include only students with both time points completed, yielding 180 matched cases. Data cleaning procedures addressed missing item responses: for the Attitudes Toward Psychiatry-30 (ATP-30) scale, total scores were prorated if a student answered at least 24 out of 30 items (missing items were imputed by averaging that student’s completed items). Similarly, for the Big Five personality subscales, mean trait scores were computed if a participant had completed most of the items for that trait, to maximize use of available data. Cases with substantial missing data were excluded from specific analyses as needed.

Descriptive statistics were obtained for all variables (calculating mean and standard deviation for continuous measures, and frequency and percentage for categorical measures). Internal consistency reliability was assessed using Cronbach’s α for the ATP-30 attitude scale (at both pre- and post-clerkship) and for each BFI-44 personality trait subscale. To evaluate change in attitudes toward psychiatry over the clerkship, we conducted a paired-samples t-test comparing ATP-30 total scores before and after the rotation. The magnitude of the pre-post change was expressed as an effect size using Cohen’s *d*.

To examine potential moderators of attitude change, we performed linear regression-based moderation analyses. In these models, the post-clerkship ATP-30 score (continuous) was the outcome variable, the pre-clerkship ATP-30 score was included as a predictor, and an interaction term between the pre-clerkship attitude and the moderator of interest (e.g. a personality trait score or role model exposure) was added. Each moderator (the five personality trait scores and the two role model variables) was tested in a separate regression model. For each model, the ATP-30 pre-clerkship score and the moderator were included as main-effect predictors along with their interaction term (ATP-30 pre × moderator). To facilitate interpretation, continuous predictors (baseline ATP-30 score and the personality trait scores) were mean-centered prior to computing interaction terms.

In a second set of moderation models, we added a group of covariates to adjust for potential confounding factors. These covariates included age, gender, nationality (Omani vs. non-Omani), region of residence (Muscat vs. other regions), academic performance (undergraduate GPA), personal history of mental illness, family history of mental illness, any prior contact with mental health services, and whether the student considered psychiatry as their first, second, or third career choice at baseline. Prior contact with mental health services refers to any clinically significant encounter with a specialized mental health service provider for evaluation, diagnosis or treatment (Mental Health Atlas 2020^28^). The inclusion of these covariates allowed us to test whether any observed moderation effect was independent of these background characteristics.

All regression models were estimated using ordinary least squares (OLS) linear regression. Because certain regression assumptions (such as homoscedasticity of errors) may be violated in our data, we obtained heteroscedasticity-consistent (HC3) robust standard errors for all regression coefficients to ensure valid inference. Statistical significance was evaluated at the 0.05 level (two-tailed). For the set of multiple interaction tests, we applied a False Discovery Rate (FDR) adjustment (Benjamini–Hochberg procedure) to control for type I error due to multiple comparisons; we report the corresponding adjusted q-values for the interaction terms. Model fit was evaluated with the R-squared (R²) statistic, representing the proportion of variance in post-clerkship attitude scores explained by each model. Exploratory sex-stratified analyses were also conducted in response to reviewer feedback. Welch’s t-test was used for continuous variables, including ATP-30 scores and Big Five personality traits, while chi-square or Fisher’s exact tests were used for categorical variables, as appropriate. Influence diagnostics for the adjusted resident role-model moderation model were examined using Cook’s distance.

All analyses included checks of model assumptions (normality of residuals, linearity, and homogeneity of variance), and no serious violations were noted after using robust error estimates.

### Ethics

The ethical approval for this study was obtained from the Sultan Qaboos University Medical Research Ethics Committee on 27th October 2023, with approval number MERC 2910.

The study abided by the recommendation of the Declaration of Helsinki. Written informed consent was obtained from each participant. Their participation was voluntary, and they held the right to withdraw from the study at any time for any proposed reason and their withdrawal did not affect them in any way.

### Confidentiality

All data relating to the participants remained confidential and was shared only with the research team. Unique Passwords and identifiers were utilized for computer-based data entry. Screening forms, assessment files of each subject, and identification code list were kept in a digitally locked file on a password-secure desktop.

## Results

### Sample characteristics and descriptive statistics

With a response rate of 90%, the matched cohort consisted of 180 medical students (57.8% female) with mean age of 22.70 years (SD^9^ = 0.82). The vast majority (96.7%) were Omani nationals, and 22.2% were from the Muscat region. Regarding prior exposure to mental health issues, 18.3% of the students reported a personal history of mental illness, 30.0% had a family member with mental illness, and 16.1% had previously accessed mental health services. At baseline, psychiatry was the first-choice future specialty for 8.9% of students (with 16.7% and 43.9% of students listing it as their second or third choice, respectively). After completing the psychiatry clerkship, nearly all students agreed that their instructors were good role models: 95.0% of students agreed that the psychiatry residents were good role models, and 97.8% agreed that the attending psychiatrists were good role models. This indicates a substantial ceiling effect on the role-model items (almost all students rated them positively; see Table 1 for details).

### Personality profile and sex differences

The personality profile of the sample showed the highest mean score for agreeableness (M = 3.89, SD = 0.47), followed by conscientiousness (M = 3.52, SD = 0.47), openness (M = 3.47, SD = 0.47), extraversion (M = 3.07, SD = 0.60), and neuroticism (M = 2.87, SD = 0.68). In exploratory sex-stratified analyses, female students had higher baseline ATP-30 scores than male students (107.85 ± 13.32 vs. 102.68 ± 12.27; Welch t = 2.69, *p* = .008) and higher post-clerkship ATP-30 scores (112.70 ± 13.12 vs. 107.21 ± 13.94; Welch t = 2.67, *p* = .008). However, the magnitude of attitude change did not differ by sex (4.85 ± 12.68 vs. 4.54 ± 12.16; Welch t = 0.17, *p* = .866). Among the Big Five traits, only neuroticism differed significantly by sex, with higher scores among female students (2.98 ± 0.68 vs. 2.73 ± 0.65; Welch t = 2.56, *p* = .011). Extraversion, agreeableness, conscientiousness, and openness did not differ significantly by sex. Psychiatry as a first or second career choice did not differ by sex; however, female students were more likely than male students to report psychiatry as a third career choice at baseline (51.9% vs. 32.9%; χ² = 6.46, *p* = .011).

### Internal consistency

Both the attitude scale and the personality trait measures showed acceptable internal consistency. The ATP-30 attitude questionnaire demonstrated good reliability at both time points (Cronbach’s α = 0.86 pre-clerkship, and 0.88 post-clerkship). For the Big Five personality traits, Cronbach’s α values ranged from 0.57 (Conscientiousness) to 0.78 (Neuroticism). The Extraversion and Neuroticism subscales demonstrated satisfactory reliability, with α values of 0.71 and 0.78, respectively. In contrast, the Agreeableness, Conscientiousness, and Openness subscales showed lower reliability (α = 0.60, 0.57, and 0.64, respectively), on the borderline of acceptable internal consistency. The reliability statistics for each scale are summarized in Table 2.

### Pre–post change in attitudes toward psychiatry

Students’ attitudes toward psychiatry improved significantly over the course of the clerkship. The mean ATP-30 score increased from 105.66 before the rotation (SD = 13.10) to 110.38 after the rotation (SD = 13.71). This gain of approximately + 4.72 points was statistically significant (paired-samples t-test: t(179) = 5.09, *p* < .001) and corresponds to a small-to-moderate positive effect size (Cohen’s *d* = 0.38). The full pre–post comparison statistics are presented in Table 3.

### Moderation analyses

#### Unadjusted models

In the initial unadjusted moderation analyses (i.e. without covariates), most of the personality traits and role model variables did not significantly moderate the change in attitudes. Out of the seven moderators tested, only one showed a significant interaction effect with baseline attitude: the resident role model variable (whether the student agreed that residents were good role models) exhibited a significant interaction with the pre-clerkship ATP-30 score in predicting the post-clerkship score. Specifically, the interaction term for ATP-30 pre × resident role model was significant (*b* = − 0.885, *p* = .025). This suggests that the influence of baseline attitude on final (post-clerkship) attitude differed depending on whether the student had a positive resident role model experience. None of the Big Five personality trait scores produced a significant interaction effect (all interaction p-values > 0.29 in these models), and the attending psychiatrist role model variable also did not show a significant interaction. The lack of an attending role model effect is unsurprising given the extreme ceiling in that item (almost all students rated attendings as good role models, providing minimal variance for analysis). The unadjusted moderation results for all moderators are summarized in Table 4.

### Adjusted models (with covariates)

After including the background covariates in the models, the pattern of results remained largely the same. Crucially, the moderation effect involving the resident role model persisted as statistically significant in the adjusted model (interaction *b* = − 0.953, *p* = .004). This interaction effect also passed the multiple-comparison adjustment threshold (FDR-adjusted q = 0.031), indicating that it is a robust finding even after controlling for the number of tests (see Table 5). In contrast, none of the interactions involving the Big Five traits or the attending role model were significant in the covariate-adjusted models (all adjusted interaction p-values > 0.20). The inclusion of the covariates did not substantially change the variance explained by the models; for instance, the model including the resident role model interaction and all covariates explained about 45.3% of the variance in post-clerkship attitudes (R² 0.453).

To interpret the significant interaction between baseline attitude and the resident role model, we probed the conditional effects at different levels of baseline attitudes. This analysis showed that for students who began the clerkship with relatively low initial ATP-30 scores (one standard deviation below the mean), having a positive resident role model was associated with a much higher post-clerkship attitude score compared to not having a resident role model. Specifically, among those with low baseline attitudes, students who agreed that residents were good role models scored about 27.5 points higher on the ATP-30 at post-clerkship than those who did not have a resident role model. In contrast, for students who started with very high baseline attitudes (one standard deviation above the mean), the difference in post-clerkship ATP-30 scores between those who had a resident role model and those who did not was small (on the order of 2–3 points) and not statistically significant. In practical terms, this finding indicates that resident role models had the greatest impact on students who initially held fewer positive attitudes toward psychiatry.

### Covariate effects

In the fully adjusted model focused on the resident role model interaction (see Table 6), most of the demographic and background covariates were not significant predictors of post-clerkship attitudes when controlling for other factors. The only covariate that showed a significant effect was prior contact with mental health services: students who had any prior personal engagement with mental health services scored about 4.55 points lower on the post-clerkship ATP-30, on average, compared to those with no prior contact (b = − 4.553, *p* = .047, 95% CI [–9.08, − 0.02]). No other covariates (such as age, gender, GPA, or personal/family history of mental illness) had a significant association with post-clerkship attitude scores in the adjusted model.

### Sensitivity Analysis

We conducted a sensitivity check concerning the Conscientiousness trait as a moderator. Using conventional OLS regression output (which assumes homoscedastic errors), the interaction between baseline ATP-30 and Conscientiousness was statistically significant in both the unadjusted and adjusted analyses (unadjusted interaction p 0.007; adjusted p 0.003). However, with the more robust HC3 standard errors, this interaction effect was no longer significant (*p* > .20 in both Tables 4 and 5). In other words, the evidence was sensitive to the statistical assumptions. Given that the significance of this effect did not hold under robust error for Conscientiousness as a moderator estimation, we did not interpret Conscientiousness as a reliable moderator in our primary results.

### Sensitivity power analysis

A sensitivity power analysis for the paired pre-post ATP-30 comparison was conducted. With *N* = 180, α = 0.05, and 80% power, the study could detect a minimum paired-samples effect of approximately dz = 0.21. Using the observed standard deviation of the ATP-30 difference scores (SD = 12.43), this corresponds to an approximate minimum detectable change of 2.61 ATP-30 points. The observed mean change of 4.72 points exceeded this threshold. However, this statement applies to the main pre-post comparison and not to the sparse subgroup analyses involving role-model ratings.

**Table 1:** Sample characteristics and descriptive statistics (*N* = 180).

Variable	M (SD)	*n* (%)
Age (years)	22.70 (0.82)	—
GPA (coded 2.25–3.75)	3.10 (0.37)	—
ATP-30 total (pre-clerkship, range 30–150)	105.66 (13.10)	—
ATP-30 total (post-clerkship, range 30–150)	110.38 (13.71)	—
ATP-30 change score (post – pre)	4.72 (12.43)	—
Extraversion (score 1–5)	3.07 (0.60)	—
Agreeableness (score 1–5)	3.89 (0.47)	—
Conscientiousness (score 1–5)	3.52 (0.47)	—
Neuroticism (score 1–5)	2.87 (0.68)	—
Openness (score 1–5)	3.47 (0.47)	—
Female	—	104 (57.8%)
Omani nationality	—	174 (96.7%)
Muscat region (residence)	—	40 (22.2%)
Personal history of mental illness	—	33 (18.3%)
Family history of mental illness	—	54 (30.0%)
Prior contact with mental health services	—	29 (16.1%)
Psychiatry as 1st career choice (baseline)	—	16 (8.9%)
Psychiatry as 2nd career choice (baseline)	—	30 (16.7%)
Psychiatry as 3rd career choice (baseline)	—	79 (43.9%)
“Residents were good role models” (post)	—	171 (95.0%)
“Attendings were good role models” (post)	—	176 (97.8%)

*M* = mean; *SD* = standard deviation; ATP-30 = Attitudes Toward Psychiatry 30-item scale. For ATP-30, total scores were prorated when minor item-level missingness occurred (at least 24 of 30 items answered). Big Five trait scores are the mean of the item ratings on a 1–5 scale for each trait (from the BFI-44 questionnaire). GPA = Grade Point Average (here coded by category midpoints: 2.25, 2.75, 3.25, 3.75). Percentages for categorical variables are calculated out of *N* = 180.

**Table 2:** Internal consistency of study measures (Cronbach’s α).

Scale	Cronbach’s α	*n* (complete cases)	k (items)
ATP-30 (Pre-clerkship)	0.86	146	30
ATP-30 (Post-clerkship)	0.88	145	30
BFI-44 Extraversion	0.71	161	8
BFI-44 Agreeableness	0.60	162	9
BFI-44 Conscientiousness	0.57	158	9
BFI-44 Neuroticism	0.78	162	8
BFI-44 Openness	0.64	162	10

Cronbach’s α values were computed using cases with complete data for all items on the given scale (n indicates the number of students with no missing item in that scale). BFI-44 = 44-item Big Five Inventory. *k* = number of items in the scale.

**Table 3:** Pre–post change in ATP-30 scores (paired-samples t-test, *N* = 180).

	Pre-clerkship M (SD)	Post-clerkship M (SD)	Mean Difference	95% CI of Difference	t(df)	p-value	Cohen’s d
ATP-30 total score	105.66 (13.10)	110.38 (13.71)	+ 4.72	[2.89, 6.55]	5.09 (179)	< 0.001	0.38

CI = confidence interval; df = degrees of freedom. Cohen’s d for paired samples is calculated using the mean of the difference scores divided by the standard deviation of those difference scores. By conventional benchmarks 0.38 represents a small-to-moderate effect size.

**Table 4:** Unadjusted moderation models (HC3 robust SEs): Interaction of baseline attitude (ATP-30 pre) with each moderator, predicting ATP-30 post (*N* = 180).

Moderator	Interaction b (robust SE)	T	*p*	FDR q	Model *R*²
Extraversion	–0.097 (0.223)	–0.433	0.665	0.665	0.332
Agreeableness	–0.193 (0.277)	–0.696	0.486	0.665	0.342
Conscientiousness	–0.339 (0.323)	–1.051	0.293	0.665	0.357
Neuroticism	0.094 (0.133)	0.706	0.480	0.665	0.330
Openness	–0.186 (0.374)	–0.497	0.619	0.665	0.335
Resident role model	–0.885 (0.395)	–2.239	0.025	0.176	0.410
Attending role model	3.789 (7.808)	0.485	0.627	0.665	0.357

*b*= unstandardized interaction coefficient (for ATP-30 pre × moderator). SE = robust (HC3) standard error of the interaction term. FDR q = False Discovery Rate adjusted p-value for the interaction (adjusted for the seven tests in this table). Each model includes baseline ATP-30 score, the moderator, and their interaction (no covariates). Model R² is the proportion of variance in ATP-30 post explained by the model.

**Table 5:** Adjusted moderation models (HC3 robust SEs): Interaction terms with covariates included (*N* = 179–180).

Moderator	Interaction b (robust SE)	t	*p*	FDR q	Model *R*²
Extraversion	–0.135 (0.240)	–0.564	0.573	0.606	0.383
Agreeableness	–0.147 (0.283)	–0.521	0.603	0.606	0.388
Conscientiousness	–0.381 (0.317)	–1.202	0.229	0.606	0.413
Neuroticism	0.112 (0.135)	0.830	0.406	0.606	0.379
Openness	–0.205 (0.378)	–0.542	0.588	0.606	0.385
Resident role model	–0.953 (0.335)	–2.844	0.004	0.031	0.453
Attending role model	4.230 (8.197)	0.516	0.606	0.606	0.403

All models include baseline ATP-30 score, the moderator of interest, their interaction term (ATP-30 pre × moderator), and the following covariates: age, gender, nationality, region, GPA, personal mental illness history, family mental illness history, prior mental health service contact, and baseline psychiatry career-choice (first/second/third option). Interaction effects are evaluated with HC3 robust standard errors. *b*= interaction coefficient (unstandardized); robust SE in parentheses. FDR q = False Discovery Rate adjusted p-value (for seven comparisons). Model R² is the variance explained by the full model. Sensitivity note: Using conventional OLS standard errors (not shown), the ATP-30 × Conscientiousness interaction was significant in these models (unadjusted p 0.007; adjusted p 0.003), but with robust SEs it was not significant (as shown above). Therefore, the Conscientiousness interaction was not interpreted as a reliable effect.

**Table 6:** Fully adjusted regression model for moderation by resident role model (HC3 robust SEs, *N* = 180).

Predictor	Interaction b (SE)	t	*p*	95% CI for b
Intercept	132.542 (30.422)	4.357	< 0.001	[72.476, 192.608]
ATP-30 pre (centered)	1.556 (0.332)	4.693	< 0.001	[0.902, 2.211]
Resident role model (Yes = 1)	15.017 (5.001)	3.003	0.003	[5.142, 24.892]
ATP-30 pre × Resident role model	–0.953 (0.335)	–2.844	0.004	[–1.615, − 0.291]
Age (years)	–1.200 (1.144)	–1.049	0.294	[–3.458, 1.059]
Female (1 = yes)	2.146 (1.983)	1.082	0.279	[–1.769, 6.061]
Omani nationality (1 = yes)	3.016 (3.752)	0.804	0.421	[–4.391, 10.424]
Muscat region (1 = yes)	–0.419 (2.088)	–0.201	0.841	[–4.542, 3.704]
GPA (numeric code)	–3.843 (2.304)	–1.668	0.095	[–8.391, 0.706]
Personal history of mental illness (1 = yes)	–0.617 (2.334)	–0.264	0.792	[–5.225, 3.992]
Family history of mental illness (1 = yes)	1.129 (2.097)	0.538	0.590	[–3.012, 5.270]
Prior mental health services contact (1 = yes)	–4.553 (2.295)	–1.984	0.047	[–9.083, − 0.023]
Psychiatry as 1st career choice (baseline)	1.263 (3.835)	0.329	0.742	[–6.309, 8.835]
Psychiatry as 2nd career choice (baseline)	–3.394 (2.629)	–1.291	0.197	[–8.584, 1.797]
Psychiatry as 3rd career choice (baseline)	–0.925 (1.754)	–0.527	0.598	[–4.389, 2.539]

*b* = unstandardized regression coefficient; SE = robust standard error; CI = confidence interval. This table displays the coefficients from the full model testing moderation by resident role model on post-clerkship ATP-30 scores. The baseline ATP-30 score was mean-centered prior to computing the interaction term. Model R² = 0.453 (approximately 45.3% of variance in post-clerkship attitudes explained). Continuous predictors (baseline ATP-30 and GPA) were mean-centered; binary predictors are coded 0 = no, 1 = yes.

Statistically, the negative interaction indicates that the presence of a resident role model attenuates the association between baseline attitudes and the outcome; conceptually, this suggests that role modelling reduces reliance on pre-existing attitudes by providing experiential and relational scaffolding that promotes more uniform outcomes across learners.

Because the resident role-model variable showed a strong ceiling effect, we examined the cell sizes and influence diagnostics. A total of 171 students endorsed residents as good role models, while only 9 did not; for attending psychiatrists, 176 students endorsed them as good role models and 4 did not. Cook’s distance was examined in the fully adjusted resident role-model model. The maximum Cook’s distance was 0.251, and no observation exceeded the conventional Cook’s D > 1 threshold. However, 14 observations exceeded the more conservative 4/N screening threshold. Excluding the single most influential observation did not remove the interaction effect (b = − 0.894, HC3 SE = 0.346, *p* = .010). Excluding all observations above the 4/N threshold also retained the interaction (b = − 1.522, HC3 SE = 0.545, *p* = .005), although this sensitivity model left only four students in the negative resident role-model group. Therefore, the interaction was statistically retained but should be interpreted cautiously because the comparison group was small.

## Discussion

### Summary of findings

The primary outcome of the analysis demonstrated a statistically significant change of small-to-moderate magnitude (*d = 0.38*) in the attitude toward psychiatry of medical students following an 8-week psychiatric clerkship, however, this change showed no significant association with baseline personality traits. Our findings align with global research, a Chinese study reported similar gains in ATP-30 scores after a comparable rotation, however, this change in attitude did not translate practically to career outlooks^29^. Despite improved attitudes, only 8.9% of students considered psychiatry a first career choice. This dissonance between positive ATP-30 scores and career selection indicates that attitude change alone is insufficient to drive specialty selection^7,30^. Oman reflects this trend; where despite the field’s relative popularity among specialty choices^31^, the country continues to face a scarcity of practicing psychiatrists to meet the increasing mental health needs of the population^1^.

### Role models and social learning theory

The improvement in ATP-30 is consistent with Social Learning Theory, which posits that the acquisition of behaviors and attitudes is done through observation and imitation^14^. Throughout their rotation, medical student’s observed complex interactions between psychiatrists and their patients. Witnessing the efficacy of psychiatric treatment, the positive attitude of the psychiatrist toward their discipline and the intellectual novelty of daily practice was associated with more positive attitudes and a reduction in negative stereotypes through experiential evidence, as students seek to imitate their mentors. Furthermore, the rotation aids in recognizing that psychological issues are prevalent even in general medicine, in this way students develop a broader awareness that helps sustain these favorable attitudes long-term^32^.

The most notable finding beyond the main effect was the significant interaction between resident role-model ratings and baseline attitudes (b = − 0.953, *p* = .004, FDR q = 0.031). However, this finding should be interpreted cautiously because the role-model item was measured post-clerkship alongside the outcome. Therefore, reverse causation cannot be ruled out; students whose attitudes improved may have retrospectively rated residents more favorably. Although the interaction coefficient was negative, this reflects a buffering effect whereby resident role models attenuated the impact of baseline negative attitudes and final outcomes. Specifically, students who entered the rotation with less favorable attitudes appeared to show the greatest improvement when they endorsed positive resident role models. The estimated conditional difference was approximately 27.5 ATP-30 points at low baseline attitude levels; however, this estimate should be interpreted cautiously because it was derived from a small comparison group of students who did not endorse residents as good role models. Therefore, the finding is best understood as suggestive evidence that residents may be particularly important for initially skeptical students, rather than as a precise estimate of effect size. Residents operate on a near-peer level^33^, occupying a position that is simultaneously proximal enough to be relatable yet sufficiently advanced to be credible. This positioning is theoretically significant and educationally strategic: within the Social Learning Theory, observational learning is most potent when the model is perceived as similar to the observer yet demonstrably successful^14^. Residents are the embodiment of this theoretical operator, where they have recently navigated the same uncertainties facing students, making their professional choices and coping strategies more legible and transferable than those of senior consultants. Their emotional accessibility provides a different learning experience; one where the students connect to via identification rather than distant admiration, which may be consequential for students who enter the rotation skeptical. In contrast, attending psychiatrists, despite being valuable as examples of clinical mastery, operate at a greater social and professional distance, which may explain the absence of a comparable moderating effect in our data despite the universal positive rating for both. Conversely, the absence of a moderating effect for attending psychiatrists is most likely explained by a ceiling effect as almost all students rated them positively (97.8%) leaving little variability for analysis.

### Personality traits

The absence of significant personality moderation in our study warrants careful interpretation. A non-significant moderation effect does not imply that personality traits are irrelevant to psychiatric career attitudes; rather, it suggests that, within the specific context of a structured 8-week clerkship, personality trait differences did not produce differential rates for attitude change. This is an important distinction. Personality traits, particularly openness to experience and agreeableness, have been associated with a greater interest in psychiatry in previous research^34^ and may influence student’s baseline attitudes toward the specialty. Research among health care students has similarly shown that agreeableness and openness shape how previous contact with mental illness translates into change in attitudes, suggesting that these traits influence not only baseline dispositions but also the processing of clinical exposure^35^. Meanwhile, the clinical experience itself modulates or reinforces those pre-existing traits. The present findings are thus better read as evidence that structured clinical exposure attenuates the moderating role of personality during this specific period, rather than as evidence that personality is without consequence for psychiatric attitudes more broadly.

One theoretical framework that may account for this pattern is Mischel’s Situational Strength theory, which proposes that the influence of personality on behavior varies according to the strength of the situational context. Specifically, “Strong situations” are environments with clear behavioral expectations leaving little room for interpretation and often leading to the suppression of individual differences as people conform to the requirements of the setting^36^. A structured medical rotation, with well oriented clinical staff represents such situation, where clear professional norms limit the observed association of baseline personality traits.

This framework explains the absence of moderation by personality observed in this study, suggesting that a student’s innate personality and temperament do not persist at the cost of their ability to learn and evolve; instead, attitude change is more likely a product of the quality of the clinical experience rather than the personality of the medical student. Furthermore, personality traits remain stable throughout the medical education^37^, which means that while these traits retain their predictive validity for long-term outcomes, they are relatively poor tools at anticipating short-term attitude shifts^38^.

Alternatively, the lack of moderation could stem from Professional Identity Formation^39^. As a form of disciplinary solidarity, constituents of a field harbor a socially mediated sense of responsibility, feeling obligated to honor the tenets of their respective fields via the expression of positive attitudes. Thereby, positive appraisal of psychiatry may reflect institutional socialization and professional duty as opposed to individual personality.

Methodologically, the lack of significant moderation should be interpreted cautiously, as three BFI subscales Agreeableness, Conscientiousness, and Openness demonstrated below-threshold internal consistency in our sample (Cronbach’s α = 0.60, 0.57, and 0.64, respectively). Measurement error within these subscales may have attenuated the observed associations and reduced our ability to detect true moderation effects. In addition, the absence of significant moderation may also be partly explained by the relatively narrow distribution of personality trait scores within the study sample. Taken together, these considerations suggest that the absence of significant personality moderation in the present study is inconclusive rather than definitive.

### Prior exposure

Finally, prior contact with mental health services was associated with lower post-clerkship ATP-30 scores (b = -4.553, *p* = .047). This association did not reach FDR-corrected threshold applied to the primary interaction terms and should therefore be interpreted as preliminary finding. One possible explanation is that these students may have been jaded by previous unfavorable interactions, creating a resistance to positive shifts and potentially leading to negative reframing of the clinical encounters. This phenomenon could also be explained through the Omani context, where mental health is often framed through the lens of spirituality and religiosity^2,40^. This tension extends beyond the discomfort it may generate in students; it shapes how patients and families engage with psychiatric services, how they adhere to treatment, underscoring the need for clinicians who can integrate religious and cultural dimensions into their practice. Encountering strictly medicalized frameworks, inconsiderate of these cultural dimensions, can be unsettling for some students, and can create dissonances that medical education, as it stands, may not be well equipped to bridge^41^. While medical students remain optimal candidates for anti-stigma interventions^32^ those with prior personal or vicarious experiences may feel discouraged from voicing dissenting opinions for fear of intolerance. Consequently, these students require a more meticulous approach to address deep-seated cultural and personal questions.

### Implications

While the clerkship was highly effective in converting initially skeptical students, it was less successful in consolidating interest among those already inclined toward psychiatry, revealing a gap in student capture that requires targeted intervention. Curriculum developers should prioritize structured resident mentorship for students who begin rotations with low baseline interest. Although attending psychiatrists were not significant moderators, they were rated positively by most students, suggesting a complementary role to residents. While the residents may facilitate early attitudinal access points through relatability, attendings serve as models of professional identity and clinical expertise. A mentorship incorporating both may be particularly effective. They should also integrate longitudinal mentorship models that provide sustained and tailored guidance helping with consolidating interest and offering reparative educational experiences for students with previous contact with mental health services to address earlier negative perceptions. Clinical educators should adopt a more active mentorship role, utilizing the clerkship as an opportunity to explicitly discuss career pathways and address misconceptions about psychiatry – particularly given that our findings show structured resident mentorship to be the most potent driver of attitude change among initially skeptical students. Policy makers should invest in infrastructure and resources to support curricular interventions.

### Strengths and limitations

The prospective single-group quasi-experimental study design and use of pre- and post-clerkship assessments strengthen confidence that the observed attitude change reflects the rotation itself. However, several limitations should be considered when interpreting these findings. The single-center design, reliance on self-reported measures, and absence of a control group limit generalizability, while the ceiling effect of attending model ratings restricted the statistical analysis of their moderating role.

Of particular importance is the co-measurement of the role-model item alongside the outcome variable. Since both were assessed post-clerkship, causal inference cannot be established; students who developed more positive attitudes may have retrospectively rated residents more favorably, creating a potential halo effect. Compounding this, the role-model measure comprised only two binary agree/disagree items, limiting the variance and nuance with which role-model influence could be captured; future studies should employ validated multi-item graded scales to assess this moderator more sensitively.

In addition, both role-model variables demonstrated substantial ceiling effects, including the resident role-model item. Although the resident interaction remained significant in influence diagnostics, only nine students did not endorse residents as good role models. This small comparison group limits the stability of the conditional estimates and means that the large estimated difference among students with low baseline ATP-30 scores should be interpreted cautiously. These findings support further investigation of resident role modelling but should not be read as definitive causal evidence.

The observed buffering pattern among initially skeptical students warrants additional caution, as it is susceptible to regression to the mean. Students with less favorable baseline attitudes may show greater improvement at follow-up simply because their initial scores were further from the sample average, independent of any true moderating effect. Thus, some of the observed gains among initially skeptical students may be due to this phenomenon rather than resident role models alone. Related to this, attitude measurements were taken immediately post-clerkship and may not reflect long-term attitudinal persistence; longitudinal studies tracking students through to career choice are needed to establish whether the observed changes are durable.

Finally, future studies would benefit from incorporating qualitative methods to explore students’ prior perceptions of psychiatry, personal or family experiences with mental illness, and initial career expectations, which would provide richer contextual understanding of the observed quantitative changes in attitude.

## Conclusion

In conclusion, psychiatry clerkship exposure was associated with a modest improvement in medical student’s attitudes, particularly among those influenced by resident role models. Personality traits did not appear to shape short-term attitude change, highlighting the primacy of educational and relational factors over stable personality dimensions. However, these improved attitudes did not consistently translate into greater interest in psychiatry as a future career, especially among students with prior mental health exposure. While structured clinical rotations remain instrumental in shaping positive attitudes, they are not sufficient for solving the psychiatric recruitment gap. Tailored mentorship and culturally sensitive training are essential to translate favorable attitudes into career interest.

## Supplementary Information

Below is the link to the electronic supplementary material.

Supplementary Material 1

## Data Availability

Data is available from corresponding author on reasonable request.

## Declarations

### Statement of originality

The authors hereby submit our research paper, entitled “Influence of Personality Traits and Clinical Role Models on Changes in Medical Students’ Attitudes Toward Psychiatry: A Prospective Moderation Analysis” and truthfully declare that the paper is a product of our original research investigation. To the best of our knowledge and belief, it contains no material previously published or written by another person nor does it include contents that are falsified or fabricated. We understand that should it be discovered that our attestations herein are not so, we accept the right of the journal to impose the appropriate sanctions.
